# Isoflavones Inhibit Hydrogen Peroxide-Induced Angiotensinogen Secretion

**DOI:** 10.3390/ijms26094029

**Published:** 2025-04-24

**Authors:** Masumi Kamiyama, Haruna Adachi, Mau Ogiwara, Madoka Ishikawa, Shieri Inoue, Miho Iwata, Hinano Urushibata, Shiho Ono, Hiyori Kato, Tamami Iwamoto

**Affiliations:** Department of Food and Nutrition, Jumonji University, 2-1-28, Sugasawa, Niiza 352-8510, Saitama, Japan

**Keywords:** angiotensinogen, diabetic nephropathy, isoflavones, polyphenols, renin–angiotensin system

## Abstract

The renin–angiotensin system helps regulate the endocrine system in modulating blood pressure, fluid volume, and body fluid electrolyte levels. The disruption of the renin–angiotensin system can lead to kidney disease onset and progression. However, the mechanism by which kidney angiotensinogen expression and secretion induce the onset and progression of diabetic nephropathy remains unclear. In this study, we used renal proximal tubular epithelial cells, which express high levels of angiotensinogen, to examine food components that regulate angiotensinogen secretion. The renal proximal tubular epithelial cells were first treated with catalase (antioxidant), daidzein, equol (an isoflavone), a MAP kinase inhibitor, ERK, p38, or JNK and then stimulated with hydrogen peroxide. After 24 h, we collected a culture medium to perform an enzyme-linked immunosorbent assay test for angiotensinogen and cells in order to perform real-time PCR to detect angiotensinogen. We found that angiotensinogen secretion increased as the hydrogen peroxide concentration increased. Catalase, daidzein, and equol decreased angiotensinogen expression and secretion. To investigate the cell signaling mechanism involved in these effects, we assessed the contribution of the MAP kinase cascade. Our data suggest the contribution of p38 and JNK. Our study shows that, in proximal tubular epithelial cells, hydrogen peroxide stimulates angiotensinogen secretion. Isoflavones and p38 inhibited angiotensinogen secretion.

## 1. Introduction

The renin–angiotensin system (RAS) is a critical regulator of blood volume and systemic vascular resistance. The RAS is active not only in the circulatory system but also in various tissues, and little is known about its role outside of the vasculature.

Recent studies have shown that the local/tissue RAS is important in skeletal muscles [1], bones [2], the cardiovascular system [3], intervertebral disc tissues [4], the brain [5], the heart [6], the adrenal glands [7], the vasculature [8,9], and the kidney [10,11,12]. Among these, the renal RAS is unique in that all components required to generate intrarenal angiotensin II are present in both interstitial and intratubular compartments along the nephron, making it a critical local/tissue RAS [13]. The intrarenal RAS is deeply involved in the mechanism of diabetic nephropathy. We previously reported that angiotensinogen (AGT) expression is elevated in patients with diabetic nephropathy [14,15,16]. AGT is a precursor of angiotensin and an upstream factor in the RAS. Research has shown that urinary AGT is an early biomarker of diabetic nephropathy [17], and this has been confirmed by several other studies [18,19,20,21,22]. Diabetic nephropathy progresses from macroalbuminuria to microalbuminuria and hyperfiltration, decreasing the glomerular filtration rate (GFR). Albumin excretion, which reflects changes in the GFR, is the clinical measure that is most frequently used to evaluate and predict diabetic nephropathy. However, renal failure is not always caused by microalbuminuria; nephropathy may occasionally appear in patients with normoalbuminuria. Intraglomerular hypertension and hypertrophy are the main causes of early hyperfiltration in the prealbuminuric phase. The role of the tubulointerstitium has also been increasingly recognized. This may be caused by the release of cytokines with proinflammatory properties by tubular epithelial cells and the involvement of the peritubular vasculature. The RAS may be involved before microalbuminuria and cause tubulointerstitial fibrosis in people with normoalbuminuria [19].

A previous study compared the levels of four different novel urinary biomarkers in the hyperfiltration, normoalbuminuria, and microalbuminuria stages of diabetic nephropathy and found that AGT and IL-18 levels were higher in patients than in controls; moreover, they were strongly associated with prealbuminuric nephropathy. AGT showed greater discriminatory value in terms of sensitivity and specificity compared with the albumin-to-creatinine ratio, urinary albumin, and estimated GFR [18].

Another study showed that normoalbuminuric and normotensive children and adolescents with type 1 diabetes had significantly higher median urinary VEGF-A/Cr, AGT/Cr, and transferrin/Cr but no difference in nephrin/Cr and KIM-1/Cr compared to controls. Urinary albumin/Cr was positively correlated with the GFR. Urinary transferrin/Cr, AGT/Cr, and VEGF-A/Cr were significantly correlated with the albumin-to-creatinine ratio but not with the GFR or diabetes risk factors, such as HbA1c or disease duration. Thus, normoalbuminuric and normotensive children and adolescents with type 1 diabetes have elevated urinary VEGF, AGT, and transferrin levels, which may indicate the development of diabetic kidney disease before the development of albuminuria [20].

However, the mechanisms underlying the increased AGT expression and secretion observed in patients with diabetic nephropathy remain unclear.

AGT is expressed in proximal convoluted tubules, glomeruli, and casa recta [15]. AGT is the only known substrate for renin, which is the rate-limiting enzyme of the RAS. Because the level of AGT is close to the Michaelis–Menten constant for renin, renin levels and AGT levels control RAS activity, and increased AGT levels may lead to elevated angiotensin peptide levels. Research in experimental animal models and transgenic (Tg) mice has shown that AGT is involved in RAS activation [23,24].

The oxidative modification of lipids, proteins, and nucleic acids by reactive oxygen species (ROS) plays a pivotal role in a wide range of common diseases and age-related degenerative conditions. Increases in antioxidative capacity are believed to protect against oxidative damage.

Isoflavones are a type of polyphenol found in legumes, including soybeans, chickpeas, fava beans, pistachios, peanuts, and other fruits and nuts [25]. It is also found in alfalfa sprouts, peanuts, and red clover [26]. Research on isoflavones indicates that they exert antioxidant functions through their ability to scavenge free radicals, reduce low-density lipoprotein levels, decrease DNA susceptibility to oxidative stress, and boost the activity and expression of antioxidant enzymes [27]. Isoflavones have been linked to a decreased risk of cardiovascular disease, osteoporosis, endocrine-responsive cancer, and menopausal symptoms, in part because of their putative antioxidant activities. Treatment with puerarin, an isoflavone, reversed the hyperglycemia-induced activation of the AGE/receptor for the AGE (RAGE) system in a dose-dependent manner, indicating that the AGE/RAGE axis may mediate the antioxidant activity of puerarin [28].

In this study, we investigated factors that regulate AGT secretion. Based on the hypothesis that food antioxidant components affect AGT secretion by the kidney, we asked whether food-derived antioxidants inhibit the development of diabetic nephropathy by decreasing AGT secretion.

## 2. Results

### 2.1. 1,1-Diphenyl-2-picrylhydrazyl Radical-Scavenging Assay

First, we performed 1,1-diphenyl-2-picrylhydrazyl (DPPH) radical-scavenging assays with daidzein and equol. As our hypothesis is that antioxidants suppress the secretion of angiotensinogen, we first examined whether isoflavones have antioxidant properties. Daidzein and equol showed antioxidant activity (Table 1) levels of 66.3% and 69.9%, respectively.

We also performed radical-scavenging assays for genistin, glycitin, daiddin, genistain, and glycitein. These exhibited antioxidant activity levels of 63.2%, 57.6%, 60.0%, 61.0%, and 60.2%, respectively.

### 2.2. Dose-Dependent Angiotensinogen Secretion

**Figure 1:**
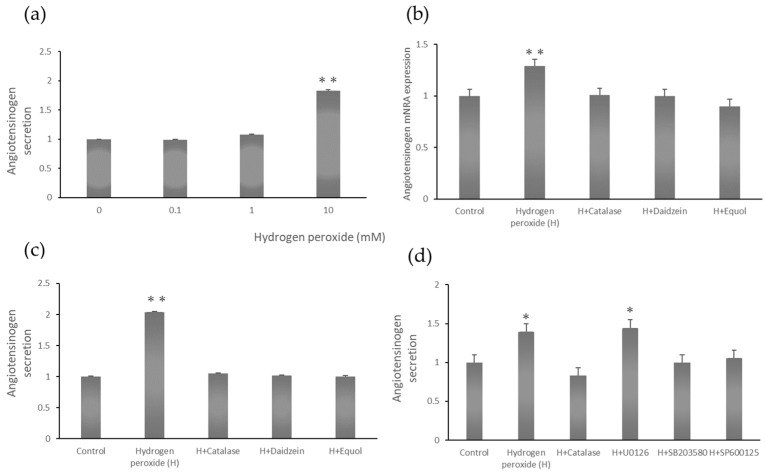
(**a**) Hydrogen peroxide increased angiotensinogen secretion in a dose-dependent manner. ** *p* < 0.01 vs. 0 mM. (**b**) Daidzein and equol decreased hydrogen peroxide-stimulated angiotensinogen (AGT) expression. Renal proximal tubular epithelial cells were treated with 300 U/mL catalase, 10 µM daidzein, or 10 µM equol for 30 min. The cells were then treated with 10 mM hydrogen peroxide for 24 h. AGT mRNA expression was measured via quantitative real-time PCR. ** *p* < 0.01 vs. control. (**c**) Daidzein and equol decreased hydrogen peroxide-stimulated AGT secretion. Renal proximal tubular epithelial cells were treated with 300 U/mL catalase, 10 µM daidzein, or 10 µM equol for 30 min. The cells were then treated with 10 mM hydrogen peroxide for 24 h. AGT secretion was measured via enzyme-linked immunosorbent assays. ** *p* < 0.01 vs. control. (**d**) Inhibiting p38 or JNK decreased hydrogen peroxide-stimulated AGT secretion. Although treatment with 10 µM of the MAP kinase inhibitor U0126 did not decrease AGT secretion, treatment with 10 µM of the p38 inhibitor SB203580 or 10 µM of the JNK inhibitor SP600125 did decrease AGT secretion. * *p* < 0.05 vs. control.

Next, we investigated whether hydrogen peroxide enhanced AGT secretion in a dose-dependent manner. The culture supernatant was collected 24 h after hydrogen peroxide addition, and 100 μL was used. AGT secretion was approximately two-fold higher in renal proximal tubular epithelial cells treated with 10 mM hydrogen peroxide compared to controls (Figure 1a).

### 2.3. Angiotensinogen mRNA Expression Stimulated by Hydrogen Peroxide

Treating renal proximal tubular epithelial cells with hydrogen peroxide enhanced AGT mRNA expression. Pretreatment with catalase decreased *AGT* mRNA expression. Catalase is an enzyme that breaks down hydrogen peroxide into oxygen and water and is present in most living organisms. Pretreatment with the isoflavones daidzein and equol significantly inhibited the increase in *AGT* mRNA induced by hydrogen peroxide in renal proximal tubular epithelial cells (Figure 1b).

### 2.4. Angiotensinogen Protein Secretion Stimulated by Hydrogen Peroxide

Hydrogen peroxide enhanced AGT protein secretion. Treatment with catalase significantly inhibited this effect. The isoflavones daidzein and equol also significantly decreased the hydrogen peroxide-induced increase in AGT secretion via renal proximal tubular epithelial cells (Figure 1c).

### 2.5. Angiotensinogen Secretion Stimulated by Hydrogen Peroxide

Hydrogen peroxide enhanced AGT protein secretion. Treatment with catalase significantly decreased this effect. The MEK inhibitor U0126 did not decrease AGT secretion, while the p38 inhibitor SB203580 and the JNK inhibitor SP600125 did decrease AGT secretion (Figure 1d).

## 3. Discussion

Diabetic nephropathy is the most common cause of end-stage renal failure, accounting for 45% of patients who start dialysis [29,30]. The RAS plays a role in the development and progression of diabetic nephropathy [15,17,31,32]. A previous study showed that urinary AGT and albumin excretion levels are elevated in diabetic mice treated with streptozotocin (STZ) [17]. Furthermore, urinary AGT excretion and intrarenal AGT expression levels increased earlier than urinary albumin levels after STZ injection compared to control mice. These findings indicated that urinary AGT is an early biomarker of increased RAS activity in type 1 diabetes.

Hyperglycemia results in the generation of ROS and attenuates antioxidative mechanisms through the non-enzymatic glycation of oxidant enzymes [33]. Another study showed that high glucose levels stimulate hydrogen peroxide production by mesangial cells [34]. Superoxide (O_2_^−^), a ROS, is produced from molecular oxygen (O_2_). The mitochondrial electron transport chain and nicotinamide adenine dinucleotide phosphate oxidase in the membrane are the most important sources of ROS under physiological and pathological conditions. Furthermore, catalase overexpression attenuated intrarenal AGT expression in an animal model of diabetes [35]. Catalase is a highly conserved heme-containing protein that reduces hydrogen peroxide to water and oxygen and is a key factor in reducing cellular damage caused by oxidative stress. Mammalian catalase is a tetrameric 240 kDa heme-containing protein that is highly conserved in humans, rats, and mice [36]. It was reported that rCatalase overexpression in the proximal tubules of Tg mice inhibits ROS generation, the expression of Agt and proapoptotic genes, and proximal tubule cell apoptosis in diabetes [37]. This indicates that oxidative stress occurs in mouse proximal tubules in the early stage of diabetes. Consistent with this, catalase upregulation reverses oxidative stress and proximal tubule apoptosis in vivo. High glucose levels promote ROS generation in rat and mouse proximal tubule cells in vivo. In this study, we further showed that, ex vivo, high glucose levels and angiotensin II induced ROS generation in proximal tubule cells from wild-type (WT) mice. Most importantly, there was no obvious increase in ROS generation in proximal tubule cells from Tg male mice stimulated with high glucose levels or angiotensin II. Furthermore, Agt mRNA levels were significantly elevated in WT proximal tubule cells incubated with high glucose levels or stimulated with Ang II compared to those incubated in a normal glucose medium. Additionally, rCatalase overexpression completely abolished the increase in Agt mRNA in Tg proximal tubule cells treated with high glucose levels or stimulated with Ang II [37]. Western blotting confirmed the upregulation of Agt in WT, but not Tg, proximal tubule cells in response to high glucose levels or Ang II. Taken together, these observations suggest that rCatalase overexpression in Tg proximal tubule cells effectively prevents the increase in ROS generation stimulated by high glucose levels or Ang II, thereby abolishing Agt gene expression.

In our study, renal proximal tubular epithelial cells were treated with hydrogen-peroxide-stimulated AGT secretion. This effect was reduced by pretreatment with the isoflavones daidzein or equol. Hydrogen peroxide induced AGT expression in renal proximal tubular epithelial cells, with the subsequent activation of p38. The pivotal roles of certain MAP kinase pathways, which are activated via hydrogen peroxide treatment, in certain cell types and cells treated with hydrogen peroxide exhibited the activation of the ERK, p38, and JNK signaling pathways [38,39,40]. In this study, we found that p38 and JNK are involved in hydrogen peroxide-induced AGT secretion. p38 MAP kinase is a member of the MAP kinase superfamily, which transmits signals into cells in response to external stimuli, such as ROS. Together with ERK and JNK, it plays an important role in acute stress responses by regulating various downstream proteins. p38 MAP kinase activation induces apoptosis, inflammation, and fibrosis [41]. JNK and p38 MAP kinase activation is observed in many acute and chronic kidney diseases and is thought to be involved in their onset and progression. A moderate stress response is important for maintaining homeostasis, but an excessive stress response can cause disease onset. Although reducing ROS or increasing antioxidant capacity has not been shown to reduce oxidative stress, our findings suggest that AGT, a downstream molecule in the oxidative stress pathway, could be a promising therapeutic target.

In recent years, Proteolysis-Targeting Chimera (PROTAC) technology has been developed for drug development [42]. Multiple-target PROTACs target multiple proteins simultaneously and may provide effective treatment for complex diseases. Napabucasin is a STAT3 inhibitor that mainly acts on cancer stem cells, but a quantitative proteomics analysis of the napabucasin-based PROTAC molecule XD2-149 revealed that XD2-149 significantly reduces the level of ZFP91 [43]. In other words, ZFP91 is a potential anti-cancer target, and is attracting attention as an application of PROTAC in drug target discovery. As a future prospect, it is expected that drug target discovery for diabetic nephropathy will also be carried out by combining this with quantitative proteomics analysis.

These data provide novel insights into the mechanisms of intrarenal RAS activation in diabetic nephropathy. We expect our findings to contribute to the development of dietary therapies for diabetic nephropathy, which is a global health problem. It is also important to organize large-scale studies on dietary therapies, including clinical research, and to confirm the mechanism by which polyphenols inhibit diabetic nephropathy progression.

## 4. Materials and Methods

### 4.1. 1,1-Diphenyl-2-picrylhydrazyl Assay

The radical-scavenging activity of extracts was determined via DPPH assays, as described previously [44]. A total of 0.012 g of DPPH (Tokyo Kasei, Tokyo, Japan) was dissolved in 100 mL of methanol (300 µmol/L). Next, 400 µL of the DPPH solution was combined with 100 µL of isoflavone solution in a test tube. Absorbance was determined at 515 nm. The absorbance at 515 nm of a 300 µmol/L DPPH solution was set at 100%, and the percentage by which the absorbance of each isoflavone solution decreased was expressed as a percentage.

### 4.2. Cell Culture and Treatment

Human renal proximal tubular epithelial cells (Cosmo Bio, Tokyo, Japan) were cultured in a renal proximal tubular epithelial cell medium (Cosmo Bio, Tokyo, Japan) at 37 °C in a 5% CO_2_ incubator. The cells were treated with 10 mM hydrogen peroxide for 24 h and then collected for analysis. In some experiments, cells were pretreated with 300 U/mL catalase (Sigma, Tokyo, Japan); 10 µM daidzein (Wako, Osaka, Japan); 10 µM equol (Wako, Osaka, Japan); 10 µM U0126, a MAP kinase inhibitor (Merck Millipore, Tokyo, Japan); 10 µM SB203580, a p38 inhibitor (Wako, Osaka, Japan); or 10 µM SP600125, a JNK inhibitor (Wako, Osaka, Japan) for 30 min, followed by treatment with 10 mM hydrogen peroxide. After 24 h, the cells were collected for analysis.

### 4.3. Enzyme-Linked Immunosorbent Assay (ELISA)

AGT concentrations in the culture medium were measured using a human AGT enzyme-linked immunosorbent assay kit (IBL, Gunma, Japan), as previously described [44]. ELISA uses a sandwich ELISA, and the first step is to coat the ELISA plate with the capture antibody and wash off excess unbound antibody from the plate. The capture antibody was an antibody raised against the antigen of interest. Cell supernatants were added to a 96-well plate, and the antigen found in the sample, angiotensinogen, was bound to the capture antibody already coated on the plate. A detection antibody was then added, labeled with horseradish peroxidase. The detection antibody binds to the target antigen, angiotensinogen, that is already bound to the plate. The concentration of antigen in the sample was analyzed using absorbance.

### 4.4. Quantitative Real-Time PCR

RNA was extracted from human renal proximal tubular epithelial cells using an RNeasy Plus Mini Kit (QIAGEN, Tokyo, Japan) according to the manufacturer’s instructions. AGT mRNA expression was examined using a One-Step Green PrimeScript PLUS RT-PCR kit (Takara Bio, Shiga, Japan) according to the manufacturer’s instructions. Quantitative real-time PCR was performed as previously described [44]. Gene expression was normalized relative to glyceraldehyde-3-phosphate dehydrogenase (*GAPDH*) mRNA expression. The primer sequences were as follows: *AGT*, forward primer 5′-tgg aca gca ccc tgg ctt tca a-3′, reverse primer 5′-aca ctg agg tgc tgt tgt cca c-3′; and GAPDH, forward primer 5′-gtc tcc tct gac ttc aac agc g-3′, reverse primer 5′-acc acc ctg ttg ctg tag cca a-3′.

### 4.5. Statistical Analysis

Data are presented as the mean ± standard error of the mean. Data were evaluated via one-way factorial analysis of variance, with Dunnett’s test as a post hoc test. *p* < 0.05 indicated statistical significance. All computations, including data management and statistical analyses, were performed using JMP 18.1.1 software (SAS Institute, Cary, NC, USA).

## 5. Conclusions

**Figure 2:**
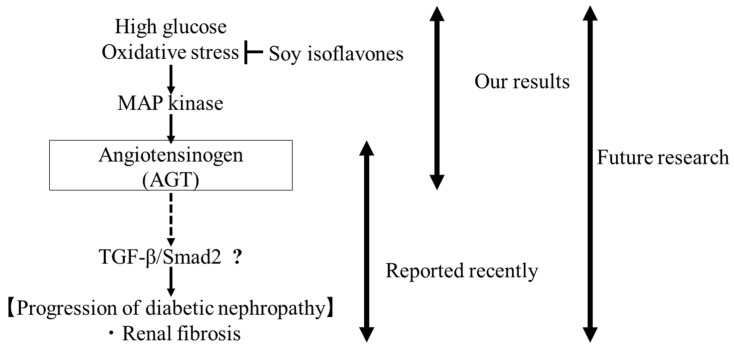
Involvement of angiotensinogen in renal fibrosis in diabetic nephropathy. “?“ means that confirmation is still required.

The purpose of this study was to determine whether antioxidant polyphenols affect AGT expression in the kidney and to clarify the mechanism by which soy isoflavones, which have strong antioxidant properties, suppress diabetic nephropathy progression through AGT expression and secretion. We found that hydrogen peroxide stimulated AGT secretion via proximal tubular epithelial cells. This effect was inhibited by pretreatment with the isoflavones daidzein or equol or by p38 or JNK inhibition. In addition, we found that p38 and JNK were involved in hydrogen peroxide-induced AGT secretion. These findings provide novel insights into the mechanisms by which intrarenal RAS activation promotes the development of diabetic nephropathy and clarify how soy isoflavone intake affects the AGT-mediated progression of diabetic nephropathy. In recent years, it has been shown that TGF-β/Smad2 is downstream of AGT and is involved in fibrosis in diabetic nephropathy [45]. In conjunction with our results, it remains to be determined whether increased AGT secretion via MAP kinases due to reactive oxygen stimulation is involved in renal fibrosis (Figure 2).

## Figures and Tables

**Table 1:** 1,1-Diphenyl-2-picrylhydrazyl radical-scavenging assays demonstrated that the isoflavones exhibited antioxidant activity.

Isoflavone	Mean ± Standard Error (%)
Daidzein	66.3 ± 0.8
Equol	69.9 ± 0.9

## Data Availability

Human renal proximal tubular epithelial cells were purchased from Cosmo Bio. The original contributions presented in this study are included in the article material. Further inquiries can be directed to the corresponding author.

## Abbreviations

AGT	angiotensinogen
DPPH	1,1-diphenyl-2-picrylhydrazyl
GAPDH	glyceraldehyde-3-phosphate dehydrogenase
GFR	glomerular filtration rate
RAS	renin–angiotensin system
ROS	reactive oxygen species
STZ	streptozotocin
Tg	transgenic
WT	wild-type
